# Are Antipsychotics for Life? Deprescribing Antipsychotic Medication in Mental Health Nursing

**DOI:** 10.1111/jan.70271

**Published:** 2025-09-26

**Authors:** Joanna M. Painter

**Affiliations:** ^1^ Rotherham Doncaster and South Humber NHS Foundation Trust Doncaster UK; ^2^ Mental Health Nursing—Sheffield Hallam University Sheffield UK

## 1 Introduction

During my early years as a non‐medical prescriber, a patient once asked me, ‘Am I a schizophrenic—and why am I four stone heavier?’ This question laid bare competing truths—the benefits of antipsychotic medication in reducing psychotic symptoms versus the significant impact those same medications can have on patients' physical health, their confidence, sense of identity and quality of life.

Globally, approximately 24 million people are living with schizophrenia, a serious mental health condition. Affecting around 0.32% of the population, the condition is typically noted to begin in late adolescence or early adulthood and is associated with substantial distress, disability and an elevated risk of premature mortality, often due to physical health comorbidities (World Health Organization 2022). However, individual diagnostic journeys are frequently complex and sometimes uncertain, with some patients and clinicians questioning the accuracy and stability of initial diagnoses over time. Many people living with a diagnosis of schizophrenia also face pervasive stigma and discrimination, compounding existing challenges.

Nurses are often uniquely placed to address these challenges, as they are frequently the first healthcare professionals to whom patients report the benefits or concerns about their medications and their side effects. This positions nurses at the forefront of difficult conversations about medication, adherence and the possibility of deprescribing.

Yet, after almost three decades in mental health nursing, now as a consultant nurse and educator, I have learned that deprescribing antipsychotic medication is rarely a straightforward process. Rather, it demands a nuanced understanding that goes way beyond any clinical trial data or protocols—it is fundamentally about people, their stories, and the difficulties of negotiating risk, benefit and choice.

## 2 Context and Current Guidelines

Guided by evidence and policy, clinical guidelines shape current practice, but questions remain about their fit with patient experiences.

Antipsychotic medication remains the recommended first‐line treatment for psychosis and schizophrenia in adults according to National Institute of Clinical Excellence (NICE 2014). These guidelines also advocate for ongoing maintenance therapy, situated within a broader package of psychosocial interventions. However, it has been suggested that secondary care is too often over‐dependent on antipsychotics as the sole treatment, with holistic and collaborative care frequently overshadowed by a biomedical focus (Kuipers et al. 2014).

Long‐term adverse effects of antipsychotics are now well documented, ranging from disabling extrapyramidal side effects to greatly increased risks of cardiometabolic disease and premature death. This knowledge has heightened global policy attention to the physical health of people living with serious mental illness, intensifying scrutiny of longstanding prescribing practices.

Within this shifting landscape, NICE and other guidelines primarily frame treatment success in terms of symptom reduction and relapse prevention. However, less attention is paid to broader outcomes such as social functioning or quality of life, which for some people might be compromised by the medications themselves.

In the UK, services for first episode psychosis commonly begin working with individuals as young as 14 years old. These services deliver holistic packages of care and good outcomes after 3 years of treatment are well evidenced. However, in practice, this means that antipsychotics are often being initiated early, and, even at low doses, are potentially committing young people to decades of pharmacological treatment. While early intervention is undoubtedly positive, this also means that antipsychotic treatment is initiated earlier in people's life course. Although guidance is detailed on the initiation and maintenance of antipsychotic medication, there remains a paucity of evidence and advice on safely reducing or discontinuing treatment (Painter 2023). As such, there remains a notable gap in guidance and research around deprescribing, which must be addressed. So, whilst it is important to accept that someone commenced on antipsychotics early in their life may need and indeed be happy to remain on that medication indefinitely, it is also reasonable to expect that others may feel that this isn't appropriate for them with the risks outweighing the benefits.

## 3 The Clinical and Academic Debate

The debate about long‐term antipsychotic use extends far beyond clinical settings. Biomedical and biopsychosocial paradigms, paternalism versus collaboration, adherence versus patient choice—these recurrent tensions inevitably shape both public policy and therapeutic interactions.

A major difficulty lies in the binary framing of debates, commonly amplified by social media discourse, which reduces complex clinical decisions to simplistic ‘for or against medication’ positions, thereby losing nuance, context and the person‐centred focus.

In response, emerging research initiatives such as those by the International Institute for Psychiatric Drug Withdrawal and the RADAR study aim to spotlight deprescribing, investigating hypotheses like slow tapering improving social functioning (Morant et al. 2023; Moncrieff et al. 2019). However, defining and measuring such outcomes remain highly personal and challenging.

## 4 Risk and Clinical Anxiety

Fear—of relapse, controversy, litigation or personal failure—is one of the greatest barriers to deprescribing, affecting both patients and clinicians. In my practice, patients often share concerns, such as, ‘They said I should stay on these tablets for the rest of my life or I'll end up back in hospital’. Similarly, clinicians voice thoughts such as, ‘he's been stable for years, let's not rock the boat’ This risk‐averse culture in mental healthcare tends to prioritise relapse prevention over autonomy and individual recovery narratives. This is understandable given how traumatic an acute relapse can potentially be for individuals and their families.

Yet, the evidence base supporting indefinite antipsychotic treatment is far from conclusive. Considerable uncertainty exists about both the full benefits of long‐term treatment and the impacts of discontinuation. Literature indicates that while deprescribing carries risks, these can often be mitigated by intensified support, gradual dose reduction and non‐pharmacological interventions, all anchored by a strong therapeutic alliance (Painter 2023). Nurses are now faced with balancing respect for patient autonomy alongside their responsibility to manage clinical risk safely. So, how do we begin to consider safe deprescribing?

## 5 Safe Deprescribing

A recent literature review identified three themes as central to safe deprescribing; these are rationales for deprescribing (side effects and diagnostic uncertainty), risk and clinical anxiety and the importance of skilled, collaborative approaches to dose reduction (Painter 2023). The PROTECT study has established future research priorities in respect of deprescribing psychiatric medication (Boland et al. 2024). Founded in multiple stakeholder experiences, this emerging body of work on safely deprescribing psychiatric medications highlights just how far the evidence is from matching the complexities faced in practice (Boland et al. 2024).

In the absence of evidence‐based protocols and guidelines, clinical negotiation and transparent risk–benefit discussion are essential. This involves regular, collaborative treatment reviews that attend as much to functioning and well‐being as to symptom control and relapse. In practice, where deprescribing is undertaken, it should be planned, gradual and accompanied by structured support that includes psychological, social and peer input as required. If we do not validate individual concerns raised about long‐term medication by having these balanced conversations, then people are more likely to reduce or discontinue medication covertly without this mitigating support in place.

## 6 Recommendations and Future Directions

- Individualize every deprescribing plan, there is no universal template.
- Hold repeated, open conversations about goals, fears and what recovery means for the person at that stage.
- Integrate psychosocial and psychological supports to address distress medication may have masked.
- Develop ‘holistic’, evidence‐based deprescribing guidelines that extend beyond initiation and maintenance, embracing the philosophy of person‐centred, holistic care.
- Promote nursing leadership in deprescribing policy development and education.

Fundamentally, underlying these recommendations is the recognition that deprescribing transcends a simple pharmacological task, constituting a holistic practice that honours personhood, autonomy and recovery.

## 7 Conclusion

Deprescribing antipsychotic medication should not merely be a clinical calculation or a ‘for or against’ decision, but a deeply individual process grounded in risk mitigation, personal recovery and therapeutic relationships. The adverse long‐term effects of antipsychotics mandate regular, collaborative treatment reviews balancing physical health, relapse prevention, functioning and patient preference. Despite its critical role in safe practice, deprescribing remains under‐researched and under‐guided. However, it is a domain where nursing's ethical and clinical leadership will be pivotal in shaping future mental health care and non‐medical prescribing practice; crucially addressing and improving patients' overall quality of life. Nursing leadership has been pivotal in progressing a culture of recovery in recent years; however, we often struggle to extend key principles of choice and autonomy to medication. Practice change is founded in supporting others to not avoid deprescribing conversations, but to embed them in care and intervention. Providing a reflective space in practice to support, educate and manage understandable anxiety is also a key first step.

Deprescribing practice must be rooted in empathy, shared dialogue and respect for the individual's narrative. For some, medication reduction or cessation brings liberation, clarity and vitality; for others, it presents an untenable risk of destabilisation.

Nurses are uniquely positioned to navigate these clinical, ethical and relational complexities by integrating evidence, lived experience and shared decision‐making. Making a commitment to patient choice as central, nursing can lead toward a prescribing practice that is as dynamic and individualized as the people it serves.

## Conflicts of Interest

The author declares no conflicts of interest.

## Data Availability

The author has nothing to report.
